# Isolation of endophytic fungi and screening of Huperzine A–producing fungus from *Huperzia serrata* in Vietnam

**DOI:** 10.1038/s41598-019-52481-2

**Published:** 2019-11-06

**Authors:** Thanh Thi Minh Le, Anh Thi Hong Hoang, Thuy Thi Bich Le, Thuy Thi Bich Vo, Dong Van Quyen, Ha Hoang Chu

**Affiliations:** 1 Institute of Biotechnology, Vietnam Academy of Science and Technology, 18 Hoang Quoc Viet, Cau Giay, Hanoi 100000 Vietnam; 2 Graduate University of Science and Technology, Vietnam Academy of Science and Technology, 18 Hoang Quoc Viet, Cau Giay, Hanoi 100000 Vietnam; 3 Institute of Genome Research, Vietnam Academy of Science and Technology, 18 Hoang Quoc Viet, Cau Giay, Hanoi 100000 Vietnam

**Keywords:** Applied microbiology, Fungi

## Abstract

Huperzine A (HupA), a natural *Lycopodium* alkaloid derived from *Huperzia serrata* (Thunb. ex Murray) Trev. plants, is a highly active acetylcholinesterase inhibitor and a key compound used for treating Alzheimer’s disease (AD). Recently, HupA has been reported in various endophytic fungi isolated from *H. serrata*. In the present study, 153 endophytic fungi were isolated from healthy tissues of *H. serrata* collected from natural populations in Lam Dong province of Central Vietnam. The endophytic fungi were identified based on morphological characteristics and Internal Transcribed Spacer sequences. Among them, 34 strains were classified into seven genera belonging to *Ascomycota*, including *Alternaria*, *Fusarium*, *Trichoderma*, *Penicillium*, *Paecilomyces*, and *Phoma*, and eight strains belonging to the genus *Mucor* (*Zygomycota*). The other strains remained unidentified. According to the results of thin-layer chromatography and high-performance liquid chromatography, only one of the 153 strains, *Penicillium* sp. LDL4.4, could produce HupA, with a yield 1.38 mg l^−1^ (168.9 µg g^−1^ dried mycelium) when cultured in potato dextrose broth, which was considerably higher than that of other reported endophytic fungi. Such a fungus is a promising candidate and alternative to presently available HupA production techniques for treating AD and preventing further memory decline.

## Introduction

*Huperzia serrata* (Thunb. ex Murray) Trev. is a rare species, and it is listed in the research program on conservation and development of rare genes in medicinal plants^1^. It is considered a medicinal herb in Western countries and is used in a wide range of functional foods sold in the market. The medicinal plant is widely known in China as Qian Ceng Ta and is used in herbal remedies for blood disorders, muscle rupture, and fever. Huperzine A, a lycopodium alkaloid first isolated from *H. serrata*, is a powerful and selective acetyl-cholinterase inhibitor (AChEI), and has attracted considerable attention because of its unique pharmacological activities and low toxicity^2–5^. Therefore, HupA is becoming a key compound in drugs for the treatment of Alzheimer’s disease^2,4^. In China, HupA was first extracted in 1948 and approved for Alzheimer’s disease (AD) treatment in the 1990s and marketed in the US as a dietary supplement, while ZT-1 has since undergone phase II clinical trials in both China and Europe^3^. The major source of HupA has been the family *Huperziaceae*; however, yields from plant extracts are extremely low. In addition, the vegetative cycle of the plants is relatively long^6^. Therefore, the production of HupA from plants for pharmaceutical manufacturing process is a major challenge. Although recent studies have demonstrated that *in vitro* cultures of *H. serrata* could provide higher quality HupA than *in vivo* cultures, the yields remain inadequate for the requirements of the pharmaceutical industry since plant tissues grow slowly in culture, and *in vitro* culture initiation requires the development of spores as starting material^7^. Consequently, the isolation endophytic fungi in HupA*-*producing *H. serrata* has been investigated by scientists with the aim of finding an alternative and increasing HupA yields for pharmaceutical manufacturing processes. Several studies have attempted to identify HupA-producing endophytic fungi from plants of the family *Huperziaceae*, particularly in China where *H. serrata* is widely distributed. HupA is biosynthesized by endophytic fungi in *Huperziaceae*, including genera such as *Acremonium*^8,9^, *Shiraia*^8,10^*, Aspergillu*s^8^*, Mycoleptodiscus*^8^*, Leptosphaeria*^8^*, Penicillium*^8^, *Trichoderma*^11^, *Colletotrichum*^12^*, Blastomyces*^13^*, Botrytis*^13^*, Ceriporia*^14^, *Hypoxylon*^14^, and so on. The some strains from *H. serrata* plants reported by far are *Acremonium* sp. 2F09P03B^9^, *Colletotrichum gloeosporoides* ES026^15^, *Shiraia* sp. Slf14^8,10^, *Cladosporium cladosporioides* LF70^16^, and *Paecilomyces tenuis* YS-13^17^.

In Vietnam, numerous studies have been conducted on endophytic fungi in plants. However, no studies on endophytic fungi in *H. serrata* have been published. In the present study, we explored and identified endophytic fungi associated with *H. serrata* in natural *H. serrata* populations in Lam Dong province in central Vietnam and determined their HupA production capacities.

## Materials and Methods

### Plant materials

The wild samples of *H. serrata* plant were collected in November 2016 and February 2017 from the natural populations at Langbiang Mountain in Lam Dong province of central Vietnam.

### Isolation of endophytic fungi

Healthy sections of *H. serrata* were sterilized using a modified version of the method described by Dobranic^18^. The samples were rinsed under running tap water and then the stems, leaves, and roots were separated and put into various beakers. They were sterilized sequentially by washing with 75% ethanol for 5 min, 10% sodium hypochlorite for 10 min, and 0.1% mercuric chloride for 2 min. Finally, the samples were rinsed in sterile distilled water four times and then each sample of the stems, roots, and leaves was cut into small pieces (0.2 to 0.5 cm) using a sterile scalpel. The small samples of each part were put in the same petri dish (9-cm diameter and each piece was spaced from 1,5 to 2 cm) containing potato dextrose agar (PDA) composed of potato extract 200 g l^−1^, glucose 10 g l^−1^, and agar 16 g l^−1^ with antibiotic streptomycin (50 µg ml^−1^) and penicillin (100 µg ml^−1^) to prevent any bacterial growth. Subsequently, the petri dishes were incubated at 28 °C in the dark and monitored every day to check the growth of endophytic fungal hyphae emerging from segments. Individual hyphal tips of the various fungi were removed from agar plates and placed on new PDA medium to check for purity using the hyphal tip method^19^ and incubated at 28 °C for five to 14 days. After purifying the isolates several times as described above, the final pure cultures were numbered and transferred to PDA slant tubes for storing at 4 °C or as spores and mycelia in 15% glycerol at 20 °C. To ensure that the surface sterilization had eliminated all epiphytic microorganisms adhering to the segments externally, the water from the final rinsing was spread on the PDA plates and incubated at 28 °C in the dark for seven days as the control. Here, the test was negative, so that no fungi grew on the control plates.

All fungal strains were grouped tentatively and dereplicated by observing their morphological and cultural characteristics, including the characteristics of the colonies on plates, aerial and substrate mycelia, spore mass color, distinctive reverse colony color, diffusible pigment, and sporophore and spore chain morphology. The similar colonies in shape, color and size were observed for hyphal length and structure with light microscopes, which to be able segregated them into distinct isolates.

Isolated fungi were preserved at the Center for Culture Collection and Genetic Recourse Conservation of Microorganisms, Institute of Biotechnology, Vietnam Academy of Science and Technology.

### Identification of endophytic fungi

The fungal colonies were cultured on plates on PDA medium at 28 °C. The hyphae or spores of the endophytic fungi were spread on slides and identified based on the cultural and conidial characteristics. Their taxonomic positions were determined using previously reported methods^20–22^. In addition, morphotypes were subjected to molecular identification methods based on the Internal Transcribed Region (ITS) sequence analyses with PCR amplification to confirm the reliability of morphological identification. The isolated fungi were inoculated into 250 ml Erlenmeyer flasks containing 50 ml potato dextrose broth (PDB) medium and cultured at 150 rpm on a rotary shaker at 28 °C for seven to 10 days. Mycelia of each fungus were obtained by centrifugation at 6000 rpm for 10 min. Subsequently, the mycelia were pulverized in liquid nitrogen, and their genomic DNA extracted using a G-spin^TM^ Total DNA Extraction Kit (INtRON, Korea) according to the manufacturer’s instructions. An rDNA region including the ITS1-5,8S-ITS2 was amplified by PCR using primers ITS1 (5′–CCGTAGGTGAACCTGCGG–3′) and ITS4 (5′–CCTCCGCTTATTGATATGC–3′) as described by White^23^. The PCR mixture (50 µl) consisted of 100 ng genomic DNA, 25 µl of 2X PCR Master Mix (Tag DNA polymerase, 0.05 U µL^−1^; reaction buffer; 4 mM MgCl_2_, and 0.4 mM of each dNTP) (Thermo Fisher Scientific), 0.5 µM of each of the primers and autoclaved double-distilled water. The PCR thermal cycling conditions included preheating at 94 °C for 5 min, followed by 35 cycles of 1 min 30 s at 94 °C, 53 °C for 1 min 30 s, 72 °C for 2 min, and a final extension step at 72 °C for 10 min. The PCR products were examined by electrophoresis on 1% agarose gel in 1X TBE buffer (40 mM Tris, 1 mM EDTA, pH 8.0) and subsequently purified using a GenneJET PCR Purification Kit (Thermo Scientific). Subsequently, the PCR products were sequenced using an AEI PRISM@ 3700 Genetic Analyzer (Thermo Fisher Scientific). The ITS sequences of the endophytic fungi, which were cleaned before submission and phylogenetic analyses, were analyzed using BioEdit and compared with the data in the National Center for Biotechnology Information database using BLAST search (http://blast.ncbi.nlm.nih.gov/Blast.cgi). Phylogenetic relationships were estimated using MEGA v6.06. The sequences were aligned with those of related fungal strains retrieved from the GenBank database using ClustalW (https://www.ebi.ac.uk/Tools/msa/clustalw2/). The phylogenetic tree was constructed using Maximum Likelihood with bootstrap values calculated from 1,000 iterations. The Kimura 2-parameter model and invariant sites (K2 + G + I) were used to estimate evolutionary distances among the species^24^.

### Preparation of endophytic fungal extracts

Each of the fungal strains (a total of 153 strains) was inoculated into 1000 ml PDB medium and cultured at 150 rpm on a rotary shaker at 28 °C for 7 to 10 days. The mycelia of the fungi were subsequently harvested by centrifugation at 6000 rpm for 10 min and dried at 45 °C overnight, and then the mycelia were ground to powder using a pestle. The powders were weighed accurately for each sample.

The extraction of HupA was according to Zhu^10^ with modifications. In the first stage, each fungal powder was moistened with 10% ammonia (1.0 ml per 1.0 g fungal powder) and then extracted by shaking with 5% hydrochloric acid for 24 hours at a rate of 30 ml per g of powder. The extracts were filtered to eliminate dregs; the water phase was rendered basic with 25% ammonia solution (pH 9) and then extracted with chloroform three times (1:1 ratio with water phase). Finally, the combined extracts were evaporated using a rotary evaporator at approximately 40–45 °C to dryness. The dry residues were dissolved in 1 ml methanol. The methanolic extracts were subjected to chromatographic separation.

### Thin-layer chromatography (TLC)

The methanolic extracts containing HupA were detected using TLC. Both TLC analysis of the methanolic extracts and the methanolic solution of HupA standard were carried out on Merck 0.25-mm silica gel plates and developed in a solvent system: chloroform: isopropanol: ethyl acetate: ammonia (4:1.5:4:0.1, v/v/v/v) by spotting on the start line of a silica gel plate (10 × 20 cm). HupA was detected with a spray reagent containing 0.5% potassium permanganate, appearing as a yellow spot. The migration of the spots was compared with reference HupA (≥98% purity, Sigma-Aldrich), to identify HupA spots.

The methanolic extract of HupA-producing strain was purified by silica gel chromatography column (0.6 × 26 cm, Merck-Germany). The 15-g silica gel 60 F254 (Merck) activated in methanol was stuffed into a column, then washed and balanced with chloroform solvent. The 1 ml of crude HupA extracts were loaded on the column and eluted with 20 ml chloroform, 100 ml of chloroform: methanol (10:0.5, v/v), and with 30 ml of chloroform: methanol (9:1, v/v). The TLC plate method was used to identify and collect the eluate containing HupA ingredients. The eluent containing HupA vacuum recovery gathered into a thick paste were crystallized in cold acetone solvent at 0–10 °C, pH 5.0–5.5, in 15 hours with three times the amount of acetone and dried to obtain purified HupA powder. The purified HupA was used for spectroscopic analyses and the AChE inhibition assay.

### High-performance liquid chromatography (HPLC)

The HupA concentration in crude fungal extract was measured using HPLC. The HPLC analysis was performed on an Agilent series 1100 HPLC system (Agilent Technologies Canada, Mississauga, ON, Canada) using a Zorbax SB-C_18_ reverse phase-column (3.0 × 150 mm) with a diode array detector. The indicator solution and the analyzed samples were filtered through a 0.45-μm filter before injection. Five microliters of the methanolic extracts were injected. The mobile phase was water (0.1% focmic acid) and methanol at a flow rate at 0.4 ml min^−1^ with a methanol gradient in the 15–100% range. A Liquid chromatography coupled to mass spectrometry (LC–MS) system was connected to Agilent OpenLAB Control Panel (Agilent Technologies) with analysis at 310 nm. Quantification was achieved using the standard curve generated from the HupA standard over a 0.01–0.7-mg ml^−1^ concentration range at which the peak area and height exhibited linear relationships with the absorbance (r^2^ = 0.9982).

Mass spectroscopy was performed on HupA samples purified as described in the TLC section previously. The Electrospray ionization combined with mass spectrometry (ESI-MS) spectrum was obtained using an Agilent 6120 Single Quadrupole LC–MS Agilent 1260 system. Purified HupA were dissolved in CDCl_3_ (Sigma, St Louis, MO) for nuclear magnetic resonance spectroscopic (NMR) analysis. The ^1^H-NMR spectra were recorded on a Bruker Avance 500 MHz spectrometer, and chemical shifts (*δ*) were expressed in ppm with reference to the tetramethyl silane signals.

### AChE inhibition

AChE inhibitory activity of the purified HupA was determined using Ellman’s method^25^. The purified HupA powder by TLC part and standard HupA, each sample was dissolved in methanol to a final concentration of 1.0 mg ml^−1^. A reactive volume of 30 µl of 2 U ml^−1^ AChE, 2810 µl of 0.1 M phosphate buffer (pH 8.0), and 30 μL of purified HupA⁄standard HupA of various concentrations (5–50 μg ml^−1^) were incubated for 10 min at 25 °C and then 100 μl of 286 μM DTNB (5′ dithiobis-2-nitrobenzoic acid) and 30 μl of 0.86 mM acetylthiocholine iodide added to the solutions. The final mixtures were incubated for 30 minutes at 25 °C. For the controls, 30 µl of 2 U ml^−1^ AChE was replaced with a similar volume of 0.1 M phosphate buffer (pH 8.0). The color developed was assessed in a microwell plate reader at 412 nm (Apel PD303, Japan). AChE activity was normalized to the control measurements. All assays were performed in triplicate and the percentage inhibition was calculated as:

$${\rm{I}} \% =({\rm{Absorbance}}\,{\rm{of}}\,{\rm{control}}-{\rm{absorbance}}\,{\rm{of}}\,{\rm{sample}})/{\rm{Absorbance}}\,{\rm{of}}\,{\rm{control}}\times 100$$

IC_50_ values were obtained from logistic regression analysis of three independent replicates. The analyses were performed using Microsoft Excel (Microsoft Corp., Redmond, WA, US).

## Results

### Isolation and morphology of endophytic fungi in *H. serrata*

**Figure 1:**
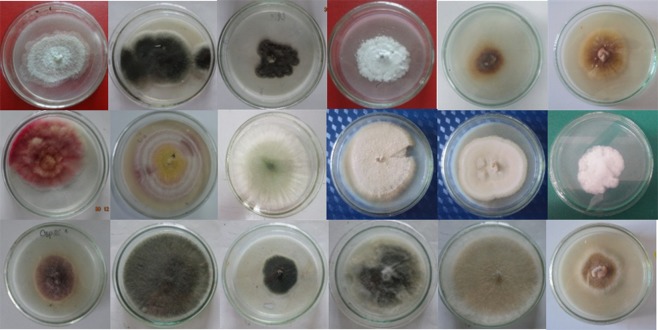
The colony characteristics of some endophytic fungi isolated from *H. serrata* tissues of Lam Dong province.

**Figure 2:**
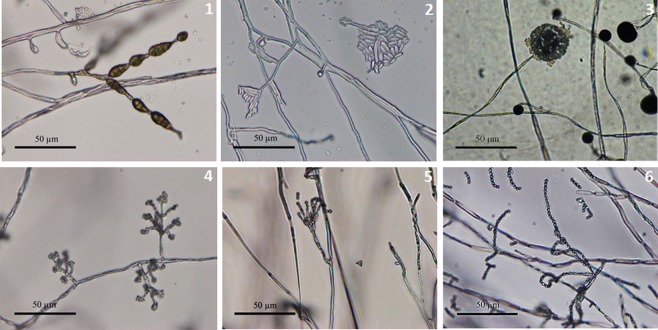
The morphological characteristics of hyphae and conidiophores by light microscope (400×) of some endophytic fungi identified belonged to genera: *Alternaria* (**1**), *Fusarium* (**2**), *Mucor* (**3**), *Trichoderma* (**4**), *Penicillium* (**5**), *Paecilomyces* (**6**).

A total of 153 endophytic fungi were isolated from 94 fresh plants and purified from 1974 tissue segments (stems, leaves, and roots) of *H. serrata* collected from natural populations at Langbiang Mountain in Lam Dong province of central Vietnam on PDA medium. The isolates were identified based on the morphology of conidia, colonies, and unique phenotypic characteristics. Among the strains, 38 strains were associated with six genera, including *Alternaria, Paecilomyces, Penicillium, Trichoderma, Fusarium* (*Ascomycota)*, and *Mucor* (*Zygomycota*) (Figs 1 and 2). The other strains could not be identified based on their morphological characteristics due to non-sporulation and were collected as unidentified fungal groups. More methods for the identification of strains are required such as specific nutrient media as well as molecular biological analysis techniques.

### Identification based on ITS sequences and phylogenetic analysis

To confirm the reliability of the morphological identification approaches, based on the ITS sequences, 5.8S rDNA region (ITS1-5.8S-ITS2), 38 strains belonged to six genera and 37 strains belonged to unidentified fungal groups. The ITS region of the strains was amplified using PCR with expected sizes ranging from 500 to 620 bp. After sequencing, identified sequences were compared with available data from the GenBank sequence database to identify genera or species. After homology searches against GenBank, the sequences of seven strains, which belonged to six genera, were classified up to the species level. Three out of seven strains (TDL69, TDL48, RDL27) exhibited 100% similarity to *Mucor circinelloides f. lusitanicus* CBS 108.17 (GenBank accession number NR126127), *Mucor fragilis* UOA/HCPF 8239 (GenBank accession number NR131304), and *Alternaria alternata* ATCC 204363 (GenBank accession number NR131316), while four strains, TDL79, LDL16, TDL1, and TDL62, were 99% similar to *Fusarium pseudensiforme* (GenBank accession number NR119942), *Fusarium keratoplasticum* FRC S-2477 (GenBank accession number NR130690), *Alternaria cerealis* CBS 119544 (GenBank accession number NR136117), and *Alternaria arborescens* CBS 102605 (GenBank accession number NR077186). Therefore, they were classified into the *Mucor*, *Alternaria*, and *Fusarium* genera and designated as corresponding strains in GenBank. The other strains of six genera were closest to the *Alternaria, Paecilomyces, Penicillium, Trichoderma*, and *Fusarium* genera based on morphology. Although some strains shared 99% sequence max identity, they only shared query cover ≤ 98% with the data available in GenBank. Among the 37 strains, which did not exhibit sporulation on PDA medium, placed into the unidentified fungal group, namely, four strains (LDL8, LDL1, LDL33, and TDL3) were classified into genus *Phoma*, and fifteen strains were closest to *Fungal endophyte* or *Fungal* (Table 1) while the others were unidentified based on their ITS sequences analysis using Blast search.

**Table 1:** Comparison of closet related species from GenBank sequence database.

Closest related species	Accession number
*Penicillium citrinum NRRL 184*	NR 121224.1
*Penicillium hetheringtonii* CBS 122392	NR 111482.1
*Penicillium tropicum* CBS 112584	NR 111485.1
*Fusarium fujikuroi CBS 221.76*	NR 111889.1
*Fusarium acutatum CBS 402.97*	NR 111143.1
*Trichoderma sp S1-1*	LT623984.1
*Trichoderma hazinum isolate FIS21*	KY378955.1
*Trichoderma hazinum strain ZG-2-2-1*	KT192387.1
*Fungal sp. Isolate E2706B*	KT996043.1
*Fungal endophyte culture-collection STRI:ICBG-Panama:TK44*	KF435852.1
*Fungal endophyte culture-collection STRI:ICBG-Panama:TK43*	KF435851.1
*Phoma herbarum strain LS2-CGS13*	KP900308.1
*Phoma herbarum strain LL2-CGL13*	KP900303.1
*Phoma herbarum strain FL10-CJL2*	KP9002441.1
*Alternaria betae – kenyensis* CBS 118810	NR_138118
*Alternaria idiriaustralis* CBS 118486	NR_136120
*Alternaria arborescens*	NR_077186
*Mucor fragilis*	NR_131304
*Mucor bainieri*	NR_103628
*Mucor circinelloides f. lusitanicus*	NR_126127
*Mucor ramosissimus*	NR_103627
*Mucor circinelloides f. janssenii*	NR_126123
*Paecilomyces sp. JCM 28097*	LC133789.1
*Paecilomyces sp. JCM 12545*	AB217857.1
*Paecilomyces formosus CBS 990.73B*	NR_149329.1
*Paecilomyces sp. BAB-4427*	KR154912.1

### Fungal taxa and distribution of endophytic fungi isolated from the natural *H. serrata* populations in Lam Dong province of Vietnam

Out of the 94 *H. serrata* plants from natural populations in Lam Dong province of central Vietnam, 153 endophytic fungi were isolated. Among the isolates, 34 strains (22.22%) were classified into the phylum *Ascomycota*, including six genera, *Trichoderma* (ten strains), *Penicillium* (eight strains), *Alternaria* (six strains), *Paecilomyces* (four strains), *Phoma* (four strains), and *Fusarium* (two strains), while eight strains (0.052%) were classified into genus *Mucor* (*Zygomycota)*. The 111 remaining strains (72.55%) were unidentified (including 15 strains classified as *Fungal endophyte* or *Fungal*). There were considerable variations in the quantities of endophytic fungi in *H. serrata* in different tissues. Eighty-four strains were isolated from the stems (54.9%), 42 from the leaves (27.45%), and 27 from the roots (17.65%) (Table 2). *Trichoderma*, *Penicillium*, *Alternaria*, *Paecilomyces*, and *Mucor* were simultaneously observed in the stems, leaves, and roots. *Phoma* were not observed in the roots, while *Fusarium* were distributed only in stems as opposed to the roots and leaves.

**Table 2:** Fungal taxa and distribution of endophytic fungi isolated from Lam Dong province of Vietnam.

Phylum	Class	Order	Family	Genus	Tissues			Total strains
					Roots	Stems	Leaves	
*Zygomycota*	*Mucormycotina*	*Mucorales*	*Mucoraceae*	*Mucor*	1	5	2	8
*Ascomycota*	*Eurotiomycetes*	*Eurotiales*	*Trichocomaceae*	*Paecilomyces*	1	2	1	4
	*Eurotiomycetes*	*Eurotiales*	*Trichocomaceae*	*Penicillium*	1	5	2	8
	*Sordariomycetes*	*Hyporeales*	*Hypocreaceae*	*Trichoderma*	1	5	4	10
	*Dothideomycetes*	*Pleosporales*	*Pleosporaceae*	*Alternaria*	1	2	3	6
	*Dothideomycetes*	*Pleosporales*	*Didymellaceae*	*Phoma*	0	1	3	4
	*Sordariomycetes*	*Hyporeales*	*Nectriaceae*	*Fusarium*	0	2	0	2
				Unidentified*	22	62	27	111
Total distinct strains					27	84	42	153
Isolation rate (%)					17.65	54.90	27.45	100
No. of samples					658	658	658	1974
No. of isolates obtained					48	102	59	209

*Unidentified strains including 15 strains described as *Fungal endophyte* sp*. or Fungal* sp. (TDL5, TDL24, TDL6, TDL49, RDL4, RDL1, LDL17, LDL23, TDL67, RDL2, TDL71, RDL4.3, TDL76, LDL5, RDL23).

### Screening of huperzine A–producing fungi based on chromatographic separation

**Figure 3:**
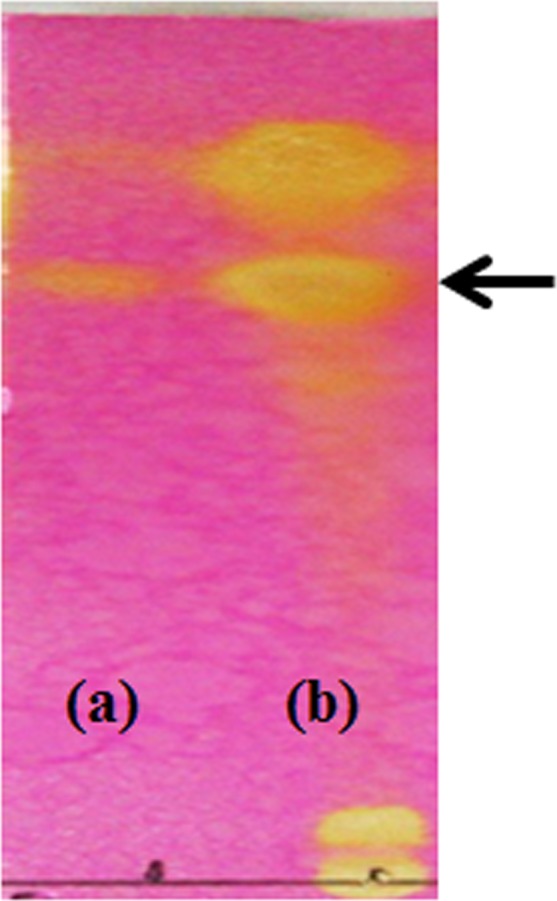
Thin-layer chromatography analysis of standard HupA (**a**) and strain LDL 4.4 HupA formation in potato dextrose broth medium (**b**) on silica gel. Arrow indicates the presence of HupA.

The 153 extracts from the fungal cultures were assessed for the presence of HupA using TLC analysis. According to the results, only one extract showed HupA production in PDB medium. The fungal compound extracted from *Penicillium* sp. LDL4.4 exhibited a retention time value of 6.9 min, which was similar to the retention time of standard HupA, in addition to a UV absorption spectrum similar to standard HupA (Fig. 3). Therefore, the *Penicillium* sp. LDL4.4 strain was selected for further characterization using HPLC.

**Figure 4:**
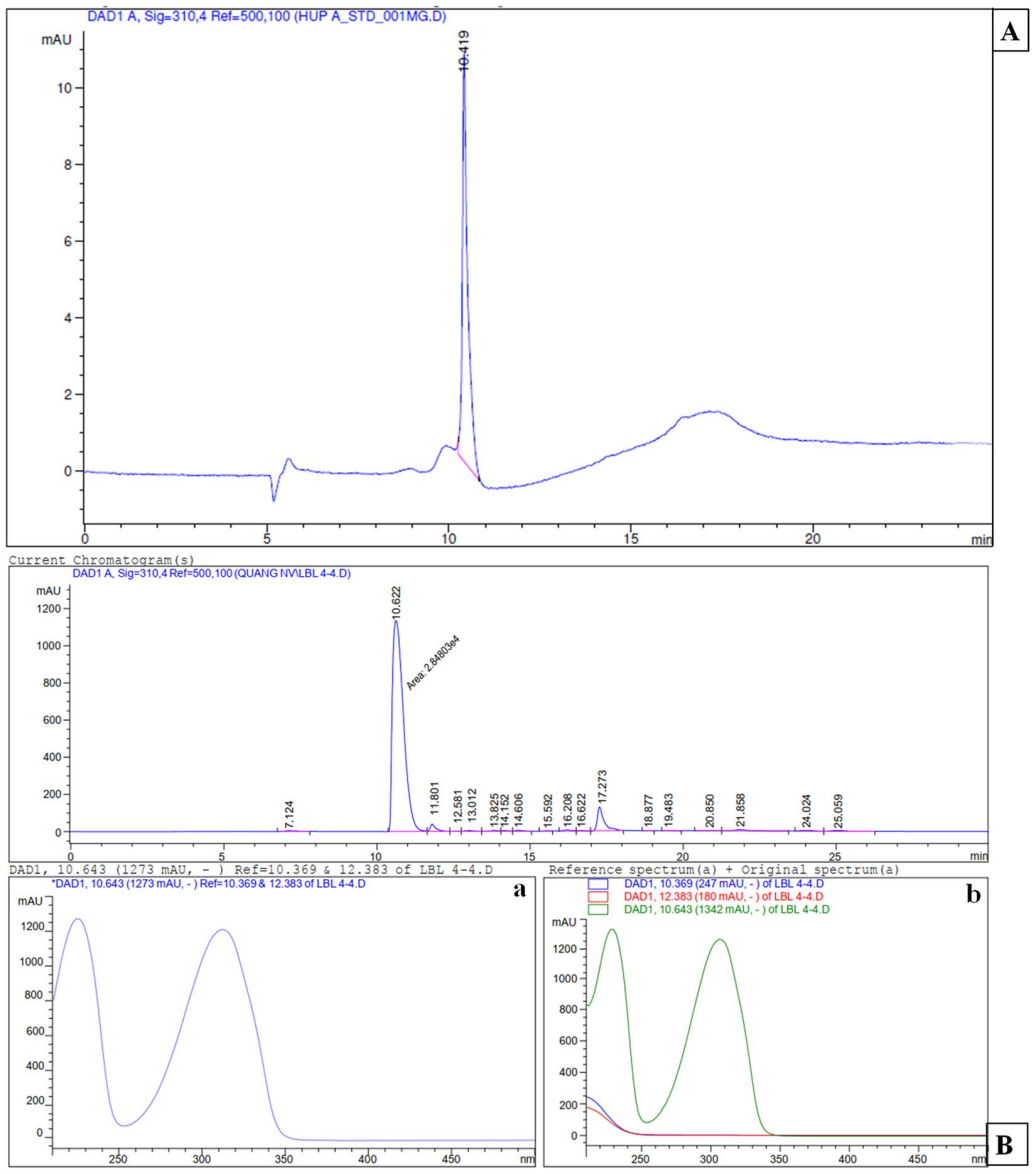
High-performance liquid chromatogram analysis of standard HupA (**A**), fungal HupA from LDL4.4 strain (**B**) and Ultraviolet absorption spectrum of standard HupA (a), fungal HupA (b). The mobile phase was 0.1% focmic acid/water (85:15, v/v) with a flow rate at 0.4 ml min^−1^. Retention time of standard HupA: 10.419 min, retention time of fungal HupA: 10.622 min.

HPLC analysis was also performed to confirm the presence of HupA in *Penicillium* sp. LDL4.4. strain. The results of HPLC analysis also confirmed the presence of HupA based on a 10.622-min retention time, which was relatively similar to the retention time of standard HupA (10.419 min). The UV absorption spectra for strain LDL4.4 extracts and standard HupA had peaks at 310 nm, with similar retention time, 10.643 min (Fig. 4). The HupA amount produced by strain LDL4.4 was quantified as 1.38 mg l^−1^ based on HPLC analysis following culture in 1 liter PDB at 28 °C with shaking at 150 rpm for five days. The weight of the dry mycelium in 1 liter PDB was 8.17 g; therefore, the HupA content in each gram of dry mycelium was 168.9 µg.

**Figure 5:**
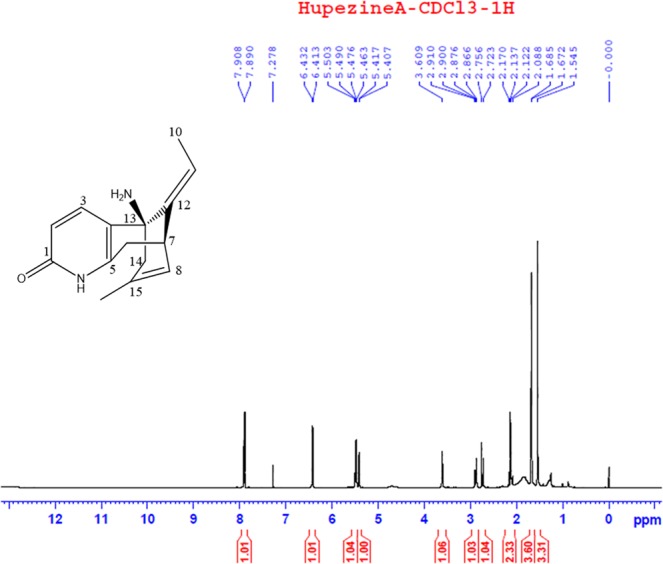
^1^H NMR spectra and chemical structure of fungal HupA extracted from strain LDL4.4.

To reveal the chemical structure of the fungal HupA isolated from *Penicillium* sp. LDL4.4, a further analysis by NMR was carried out, which showed that the ^1^H-NMR spectrum of the fungal HupA from LDL4.4 strain was consistent with the spectrum of HupA as described by Liu^26^ (Fig. 5), including ^1^H NMR (500 MHz, CDCl3), (ppm): 7.89 (1 H, d, J = 9.0 Hz, H-3), 6.41 (1 H, d, J = 9.0 Hz, H-2), 5.46 (1 H, q, J = 7 Hz, H-11), 5.41 (1 H, d, J = 5 Hz, H-8), 3.61 (1 H, brs, H-7), 2.86 (1 H, dd, J = 5 Hz, H-6a), 2.72 (1 H, brd, H-6b), 2.08 (1 H, m, H-14), 1.67 (3 H, d, J = 7 Hz, CH3-10), 1.16 (3 H, s, CH3-16). Considering the data obtained from TLC, HPLC, and MS analyses, the results demonstrated that the fungal compound from LDL4.4 strain was HupA. In addition, to the results demonstrated that *Penicillium* sp. LDL4.4 could produce HupA.

### Morphology and molecular phylogenetics of strain LDL4.4

**Figure 6:**
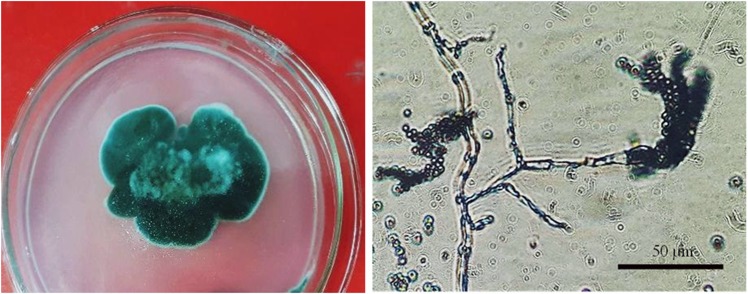
The morphological characteristics of colony, hyphae and conidiophores by light microscope (400×) of LDL4.4 strain.

*Penicillium* sp. LDL4.4 was isolated from the leaves of *H. serrata* collected from Lam Dong province in central Vietnam. The strain was characterized by colonies 4.6 cm in diameter after five days of culture in PDA medium at 28 °C. The colonies had a green color with powdery surfaces and yellow reverse coloration and white edges. The hyphae of the mycelia separated and branched into networks with phialides, reproducing by forming chains of globular spores at the ends of each of the branches (Fig. 6). The above-mentioned characteristics revealed the strain LDL4.4 belongs to the genus *Penicillium*^27^.

**Figure 7:**
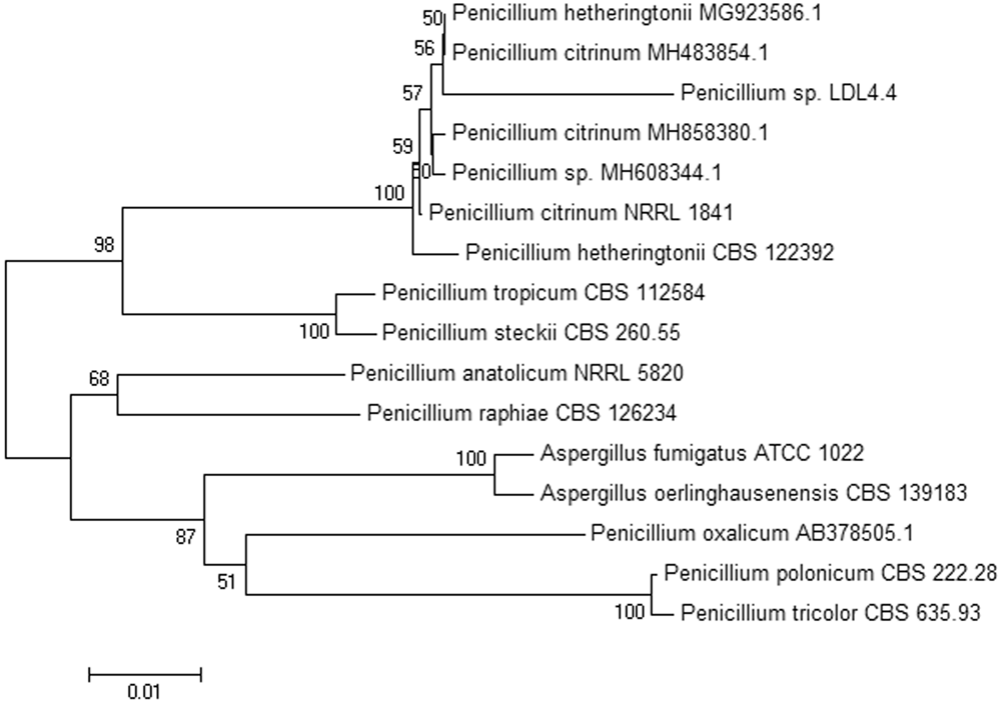
Phylogenetic tree presents the relationship of strain LDL4.4 with other related fungal species based on the sequence homologies of 5.8S rDNA sequences.

The ITS rDNA sequencing and phylogenetic analyses were used to identify the LDL4.4 strain. The ITS1-5.8S-ITS2 region was amplified using PCR with expected sizes of 531 bp. After sequencing, the sequences were compared with available data from the GenBank database. The novel sequences of the LDL4.4 strain shared were similar to the *Penicillium citrinum* NRRL184 (99%), *Penicillium hetheringtonii* CBS122392 (98%), and *Penicillium tropicum* CBS112584 (96%) sequences, while the sequence max identity shared with the above strains were 88%, 84%, and 82%, respectively, based on GenBank data. A phylogenetic relationship was established through alignment and cladistic analysis of homologous nucleotide sequences among the fungal species (Fig. 7); and strain LDL4.4 was classified into the genus *Penicillium* and designated as *Penicillium* sp. LDL4.4.

### AChE inhibition

**Figure 8:**
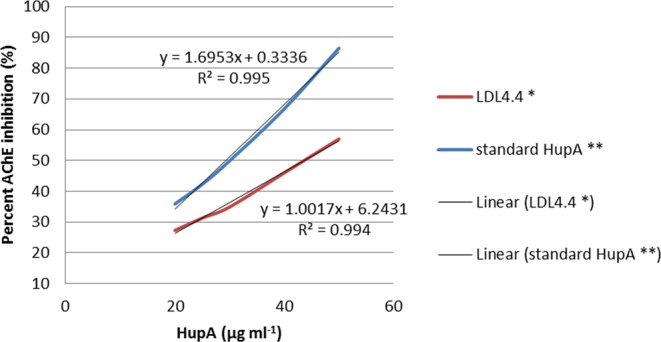
The inhibition effects (IC_50_) of purified fungal HupA from strain LDL4.4, and standard HupA *in vitro* on acetylcholinesterase activity. Values are means SEM expressed as % inhibition, *P = 0.0002, **P = 0.00015.

The AChE inhibition activity of HupA produced by LDL4.4 strain was compared with AChE inhibition activity standard HupA *in vitro* (Fig. 8). All mean values were calculated from three replicates. The IC_50_ for the purified fungal HupA was 43.68 µg ml^−1^, which was higher than the IC_50_ of standard HupA (29.29 µg ml^−1^).

## Discussion

Numerous endophytic fungi have been reported previously following isolation from *H. serrata* in various regions, particularly in China where *H. serrata* is distributed widely, including in tropical (Hainan province-southern China^28^, temperate (Hunan province-central China^29–31^ and subtropical (Anhui province-east China^32^, Guangxi province-southern China^33^, Jiangxi province^8^ and Zhejiang province^11^-eastern China, Hubei province-central China^12^, and Fujian province-southeast China^17^ regions. They represent a phylogenetically diverse range of fungal taxa (Table 3). In Vietnam, *H. serrata* are found in subtropical mountains of Lam Dong (Central Vietnam) and Lao Cai (Northern Vietnam). It is a rare medicinal plant that is being conserved. In the present study, endophytic fungi from Lam Dong province were isolated for the first time in Vietnam. The quantities of endophytic fungi from *H. serrata* varied across different plant tissues. Previous studies have reported that stems are the locations harboring the most endophytic fungi in *H. serrata*^8,32,33^. In the present study, 54.9% of the endophytic fungi were isolated from *H. serrata* stems, similar to the previous results from similar subtropical environments and culture media, including 55.6% collected in Anhui province^32^, 64.15% in Guangxi province^33^, and 47.24% in Jiangxi province^8^ from *H. serrata* stems. However, the proportions of strains isolated from *H. serrata* roots in Lam Dong province of Vietnam (17.65%) were higher than the proportions isolation in some regions of China (Anhui province^32^: 3.8%, Guangxi province^33^: 9.43% and Jiangxi province^8^: 11.02%). In addition, the endophyte species assemblages of stems were richer. It seems likely that stem tissues may provide better niche or more entry for endophytes colonization and penetration, which we need toinvestigate more extensively. There were at least seven fungal genera-*Alternaria*, *Fusarium*, *Trichoderma*, *Penicillium*, *Paecilomyces*, *Phoma*, and *Mucor* obtained from the roots, stems, and leaves of the natural *H. serrata* populations at Lam Dong. The genus *Mucor* has only been found in plants from temperate regions (Hunan province^29–31^), while *Trichoderma* have been observed from plants in Hainan province^28^ (tropical region). The six genera, including *Alternaria*, *Fusarium*, *Trichoderma*, *Penicillium*, *Paecilomyces*, and *Phoma* have been observed in *H. serrata* in subtropical regions of China, however, not all of them have been simultaneously observed in the provinces, excluding Anhui^32^. While *Fusarium*, *Trichoderma*, and *Alternaria* were observed simultaneously in Guangxi province^33^, *Penicillium* were observed in Zhejiang^11^ and Hubei^12^ provinces, and *Penicillium* and *Alternaria* have been collected from Jiangxi province^8^. Some of other genera were unidentified in the present study (Table 3). The quantitative differences of endophytic fungi and the distinctive fungal communities within *H. serrata* individuals could be attributed to the climatic and regional variation^19,34–37^. Morphological characteristics are considered conventional approaches for identifying endophytic fungi in plants. Some endophytic fungi in plants are unidentifiable because they are non-sporulating when cultured on fist isolated medium (e.g., PDA, MS)^33,38–41^. DNA sequencing analyses, including ITS, have been applied extensively for the identification of non-sporulating fungi. Some strains were recovered as fungi described previously, including *Fungal endophyte* sp. and *Fungal* sp. (Table 2). The present study could have failed to detect all endophytes within the *H. serrata* tissues because of the limitations of traditional techniques. In addition, the detection of endophytes relies on the capacity of the fungi to grow from living tissues onto the agar, or the current molecular databases are not adequately extensive and may not contain all fungal rDNA sequence information. Therefore, more effective methods of identifying more taxa, such as growing in various agars, high-throughput sequencing, denaturing gradient gel electrophoresis, terminal restriction fragment length polymorphism coupled with phylogeny analysis^42,43^.

**Table 3:** Comparison of endophytic fungi isolated from different regions.

Location and references	Isolates	Identifcation methods	Isolated and identifed fungi	Reported HupA producing fungi
Anhui province^32^	180	Morphological characteristics	*Colletotrichum*, *Trichoderma*, *Fusarium*, *Aspergillus*, *Alternaria*, *Cephalosporium* *Guignardia* *Penicillium* *Diaporthe*, *Phoma* *Verticilliun* *Phacodium*, *Paecilomyces* and mycelia sterilia	—
Hunan province^29^	4	Morphological characteristicsand ITS sequencing analysis	*Cladosporium*, *Penicillium*, *Fusarium*, and *Coniothyrium*	—
Jiangxi province^8^	127	Morphological characteristics and ITS sequencing analysis	*Colletotrichum*, *Aspergillus*, *Podospora*, *Penicillium*, *Acremonium*, *Coniothyrium*, *Paraphaeosphaeria*, *Leptosphaeria*, *Mortierella*, *Capronia*, *Chaunopycnis*, *Cladosporium*, *Botrytis*, *Shiraia*, *Alternaria*, *Mycoleptodiscus*	*Penicillium* *griseofulvum* LF146, *Penicillium* sp. SF142, *Aspergillus* *flavus* LF40, *Cladosporium* *cladosporioides* LF70, *Shiraia* sp. LF15, *Shiraia* sp. Slf14, *Acremonium* *implicatum* LF30, RF83, LF5. *Shiraia* sp. Slf14 was 327.8 µg hupA/l or 142.6 µg gdcw^−110^ *Cladosporium* *cladosporioides* LF70 was 56.8 µg l^−1^ or 39.61 µg gdcw^−1 16^
Hunan province^31^	7	Morphological characteristics	*Peziza*, *Coniothecium*, *Paecilomyces*, *Penicillium*, *Saccardia*, *Aspergillus*	—
Guangxi province^33^	53	Morphological characteristics and ITS sequencing analysis	*Glomerella, Colletotrichum, Trichoderma, Gibberella, Fusarium, Lecythophora, Coniochaeta, Rhizoctonia, Alternaria, Aspergillus, Plectosphaerella, Pestalotiopsis, Periconia, Cryptosporiopsis, Pythium, Nigrospora, Guignardia, Diaporthe, Phomopsis, Cyphellophora, Leptosphaeria, Podospora*	—
Hainan province^28^	52	ITS sequences and phylogenetic analysis	*Glomerella*, *Colletotrichum*, *Hypocrea*, *Trichoderma*, *Lecythophora*, *Coniochaeta*, *Pleurostoma*, *Chaetomium*, *Daldinia*, *Xylaria*, *Hypoxylon*, *Nodulisporium*, *Cazia* and *Phellinus*	—
Hunan province^30^	155	Morphological characteristics and ITS sequencing analysis	*Colletotrichum,* *Cladosporium,* *Sordariomycetes,* *Guignardia,* *Mucor, Neurosora,* *Penicillium,* *Aspergillus*	—
Zhejiang provine^11^	94	Morphological characteristicsand ITS sequencing analysis	*Penicillium,* *Colletotrichum,* *Acremonium,* *Shiraia,* *Leptosphaeria,* *Podospora,* *Cladosporium,* *Mortierella,* *Trichoderma,* *Paraconiothyrium,* *Arthrinium,* *Aspergillus*	*Trichoderma* *harzianum* L44 (37.63 µg/g on dry weight)
Hubei province^12^	>200	Morphological characteristics and ITS sequencing analysis	*Ilyonectria,* *Colletotrichum,* *Podospora, Pezicula,* *Elaphocordyceps,* *Chloridium,* *Paraconiothyrium,* *Cladosporium,* *Penicillium,* *Geomyces,* *Trichoderma,* *Ochroconis,* *Umbelopsis,* *Pestalotiopsis,* *Saprolegnia, Peziza,* *Clonostachys,* *Nigrospora*	*Colletotrichum* *gloeosporioides* ESO26 (1 µg/g dried mycelium). HupA yield increased from 25.47 to 32.75 µg/g CDW by optimal fermentation^7^
Fujian province^17^	201			*Paecilomyces* *tenuis* YS-13 (21 µg l^−1^)
Tis study (Lam Dong province, Vietnam - not yet reported)	153	Morphological characteristics and ITS sequencing analysis	*Alternaria,* *Paecilomyces,* *Penicillium,* *Trichoderma,* *Fusarium, Phoma,* *Mucor*	*Penicillium* sp. LDL4.4 (1.38 mg l^−1^ or 168.9 µg/g on dry weight - not yet reported)

In the present study, the first time in Vietnam to the best of our knowledge, a HupA-producing endophytic fungus LDL4.4 was isolated from *H. serrata*. The strain, isolated from the leaves of *H. serrata* obtained from the mountains of Lam Dong province in central Vietnam. The strain LDL4.4 was identified as *Penicillium* sp. based on morphological characteristics and nuclear ribosomal DNA ITS sequencing analysis. Quantitative HPLC analysis revealed that the HupA content of *Penicillium* sp. LDL4.4 was 1.38 mg l^−1^ or 168.9 µg gdcw^−1^, which were higher than those of other fungi reported previously in *H. serrata* (Table 3), such as *Acremonium* sp. 2F09P03B^9^ (8.32 µg l^−1^), *Shiraia* sp. Slf14 (327.8 µg l^−1^ or 142. 6 µg gdcw^−1^)^8,10^, *C. cladosporioides* LF70^16^ (56.8 µg l^−1^ or 39.61 µg gdcw^−1^), *C. gloeosporoides* ES026^12^ (1 µg gdcw^−1^ and HupA production in ES026 increased from 25.47 to 32.75 µg gdcw^−1^ following optimal fermentation^15^), *Trichoderma* sp. L44 (37.63 µg/g on dry weight basis)^32^ and *P. tenuis* YS-13^17^ (21 µg l^−1^) (Table 3). Previous studies have demonstrated that HupA concentrations in the leaves are higher than in the stems and roots of *H. serrata*^44^. Strain LDL4.4 was isolated from leaf tissues of *H. serrata*. The findings are consistent with the theory of Young^45^ that in the course of evolution, symbiotic endophytes developed machinery to biosynthesize and tolerate high levels of secondary metabolites to better compete and survive in association with plant tissues with medicinal properties. The results suggest that *Penicillium* sp. LDL4.4 is a promising candidate for large-scale HupA production. To satisfy commercial HupA requirements, further studies should be carried out to improve *Penicillium* sp. LDL4.4 yield using approaches such as optimal fermentation and genetic engineering to facilitate efficient HupA production using cultured endophytes. Compared to chemical synthesis and plant tissue culture methods of HupA production, endophytic fungal strains such as strain LDL4.4 would be ideal alternatives for host plants due to their relatively shorter cultivation time, ease of manipulation, and low fermentation costs. Currently, LDL4.4 strains have been preserved at the Center for Culture Collection and Genetic Resource Conservation of Microorganisms, Institute of Biotechnology, Vietnam Academy of Science and Technology, and a patent application associated with HupA production from the strain is under consideration in Vietnam (Application No. 1-2018-03576, filed on 16 October 2018).

## Conclusions

In the present study, the first in Vietnam, we isolated and assessed the diversity of endophytic fungi from *H. serrata* plants in Vietnam, and identified a novel HupA-producing fungal strain, *Penicillium* sp. LDL4.4. The production of HupA by the endophyte *Penicillium* sp. LDL4.4 is notable, since it is higher than those of other reported endophytic fungi. It is a promising candidate for a large-scale HupA production, and could be an alternative to chemical synthesis and plant tissue culture methods of HupA production and increase the amount of HupA available for the pharmaceutical industry for the treatment of AD and prevention of further memory decline and other conditions.
